# Caregiver-Implemented Lower-Intensity EMT en Español Para Autismo: A Single Case Design Study

**DOI:** 10.3390/bs16081457

**Published:** 2026-08-21

**Authors:** Natalie S. Pak, Jennifer L. Venne, Ann P. Kaiser, Tatiana Nogueira Peredo

**Affiliations:** 1 Department of Special Education, Peabody College, Vanderbilt University, Nashville, TN 37212, USA; ann.kaiser@vanderbilt.edu (A.P.K.); tatiana.peredo@vanderbilt.edu (T.N.P.); 2 Department of Communication Sciences and Disorders, University of South Florida, Tampa, FL 33620, USA; jvenne@usf.edu

**Keywords:** autism, cultural and linguistic adaptation, caregiver-implemented language intervention, treatment intensity, Spanish

## Abstract

Autism is a prevalent neurodevelopmental disability among children in the United States. Enhanced Milieu Teaching (EMT) en Español Para Autismo is a caregiver-implemented language-focused naturalistic developmental behavioral intervention specifically for young children on the autism spectrum whose families are Latino and Spanish-speaking. The current study tested a reduced intensity adaptation of EMT en Español Para Autismo using a single-case experimental design study with four caregiver–child dyads. All children demonstrated characteristics of autism and lived in low-income Spanish-speaking households. Caregivers were taught to use EMT en Español Para Autismo strategies during play with their child using a cyclical teach–model–coach–review approach. Home visits occurred once each week. Three out of four dyads completed the study. None of the caregivers demonstrated functional relations between the cyclical teach–model–coach–review approach and their use of strategies; however, caregivers did increase their use of contingent language models and time delays when intervention began. Caregiver impressions of the intervention were positive, but they varied in their perceptions of some of the strategies (e.g., limiting instructions), consistent with participants in prior studies. Overall, the reduced intensity of the intervention and long gaps between visits may have limited the effectiveness of this intervention compared to findings in prior studies. More research is needed to tailor language interventions for Latino Spanish-speaking families with autistic children, especially those with limited resources.

## 1. Introduction

Autism is a neurodevelopmental disability defined by differences in social communication and restricted or repetitive behaviors, interests, and activities (American Psychiatric Association, 2022). The prevalence of autism in young children in the United States (currently 1 in 31) has been increasing for years, especially among Latino children (Shaw et al., 2025). Caregiver-implemented evidence-based interventions are associated with positive social communication outcomes for young children on the autism spectrum (Roberts et al., 2019; Sandbank et al., 2020). These positive impacts may extend in the long term, as well. Receiving early intervention services before age three for children with developmental disabilities has been associated with improved standardized test scores in third grade, and the impact may be especially high for Latino children (Stingone et al., 2026).

Relatively few caregiver-implemented language interventions for autistic toddlers have been culturally tailored or adapted for Latino Spanish-speaking families (DuBay, 2022; Lee et al., 2025). Lee et al. (2025) identified only five experimental studies investigating culturally responsive autism interventions delivered in Spanish to children and families. However, across all languages, Lee et al. (2025) also found large and significant effects for both child and family outcomes (social communication, mental health, intervention fidelity). These effects are evidence that cultural adaptation and tailoring of autism interventions can result in effective and valuable interventions. Frameworks and tools can support cultural adaptation of existing evidence-based interventions for autistic children that were designed for English-speaking children. For example, the Cultural Adaptation Checklist and the Ecological Validity Framework upon which it is based list several dimensions for cultural adaptation, such as the language, persons, and methods (Bernal et al., 1995; Lee et al., 2024).

Naturalistic developmental behavioral interventions (NDBIs) are a class of interventions with the current best evidence among early childhood autism interventions for supporting social communication, language, and play skills (Sandbank et al., 2020; Schreibman et al., 2015). NDBIs integrate well-established principles from the fields of developmental science and applied behavior analysis (Schreibman et al., 2015). Research on cultural adaptations of caregiver-implemented NDBIs for Latino Spanish-speaking families in the United States is emerging (Gevarter et al., 2022; McGuire et al., 2025; Meadan et al., 2020; N. S. Pak et al., 2024; Pickard et al., 2024; Rollins et al., 2024). Common NDBI adaptations include translating materials into Spanish, ensuring cultural and linguistic matches between providers and families, and collaborating with community partners. For example, Pickard et al. (2024) delivered a translated version of the NDBI Project ImPACT to families via an existing outpatient early intervention service organization. Bilingual providers adapted Project ImPACT by adding education about autism, supporting families in navigating appointments, offering some sessions via telemedicine, emphasizing the benefits of bilingualism, and more. Specific strategies, such as wait time and following the child’s lead, have been less well-received by some Latina caregivers (L. M. Cycyk & Huerta, 2020; McGuire et al., 2025; N. S. Pak et al., 2024; Rollins et al., 2024).

Enhanced Milieu Teaching (EMT) en Español Para Autismo is an adaptation of EMT, a language-focused NDBI. The goals of EMT are to increase the frequency, diversity, and complexity of communication in independent and generalized contexts for young children with a variety of developmental profiles (Kaiser & Pak, in press; N. S. Pak et al., 2024). EMT en Español is a culturally adapted version of EMT for Latino Spanish-speaking caregivers and young children with language delays (Peredo et al., 2018, 2022). Adaptations included selecting language targets based on Spanish developmental sequences and family dialect, modifying the strategy of following the child’s lead so that caregivers chose the activity, and simplifying time delay and prompting procedures.

N. S. Pak et al. (2024) tested EMT en Español Para Autismo, an iteration of EMT en Español for children on the autism spectrum. The children were two 2-year-old boys with very limited spoken language (0–1 spoken words across two 20 min language samples). Core EMT en Español interaction strategies remained the same as those adapted from EMT for children with language delays (Peredo et al., 2018, 2022). Adaptations for EMT en Español Para Autismo included the introduction of augmentative and alternative communication (AAC), a phase of direct therapist intervention with the child, collaborative selection of activities and intervention materials, individualized supports for engagement, and education about bilingualism and autism. A table with examples of cultural adaptations corresponding to each dimension of the Cultural Adaptation Checklist (Lee et al., 2024) is in Supplementary Materials (Supplementary File S2). The single-case design study was a multiple baseline design across four sets of EMT en Español strategies, replicated across two dyads. These two caregivers used the strategies in observed caregiver–child interactions, and both children increased their rates of communication. Both caregivers also used aided modeling with speech generating devices with their children.

In the N. S. Pak et al. (2024) study, the intervention schedule was relatively intensive (up to three 1 h sessions per week). In addition to the two caregivers who completed the study, one caregiver could not enroll and two other caregivers dropped out. These families reported competing demands on the family’s time, such as the child starting school. The caregivers who dropped out resided in lower-income households, suggesting the high intensity of intervention was not feasible for families facing additional burdens of economic hardship. These findings indicate that the *methods* or *context* dimensions of the cultural adaptation process may not have been adequately addressed, particularly as the majority of Spanish-speaking and Latino children in the US reside in low-income households (Bernal et al., 1995; DuBay, 2022; Lee et al., 2024; The Annie E. Casey Foundation, 2025). It is critical to adapt NDBIs, so they are accessible to Spanish-speaking families of children on the autism spectrum who have competing demands and limited time.

The relation between treatment intensity and treatment effects for children on the autism spectrum is not straightforward. Applied behavior analysis approaches often recommend up to 40 h per week of direct intervention (e.g., Cohen et al., 2006). One unique aspect of NDBIs is that they emphasize the social context in which teaching episodes occur, and implementers capitalize on salient moments for teaching (Schreibman et al., 2015). Thus, a simple measure of the quantity of teaching episodes or hours of intervention may not relate directly to effects (Warren et al., 2007). In a meta-analysis of 27 NDBI studies involving children on the autism spectrum, there were no associations between treatment intensity (measured in cumulative hours) and treatment effects (Crank et al., 2021). While acknowledging the potential limitations of the dataset, Crank et al. (2021) concluded that decisions regarding intervention intensity and other features should be based primarily on the child and family needs.

Studies specifically focusing on low-intensity or low-dose caregiver-implemented NDBIs for autistic children and families have had promising effects on child communication skills (Ingersoll et al., 2017; Lane et al., 2016, 2025; Rollins et al., 2021). In a randomized control trial, Rollins et al. (2021) found that toddlers in the NDBI group who attended one 90 min home visit per week for 12 weeks made significantly greater gains on social measures compared to toddlers in the control group who attended an average of 6.65 h per week of community-based services. In a single-case experimental study, Lane et al. (2025) demonstrated that two caregivers with autistic toddlers used naturalistic language strategies when taught during 1 h weekly Zoom meetings for a brief period of time. Each meeting contained multiple 4 min intervention sessions with coaching and feedback, and there were 3–9 sessions per strategy. Although caregivers did not maintain the use of the strategies at criterion levels after intervention ended, the authors demonstrated that the rapid coaching intervention could have utility for families waiting for longer-term services or requiring additional specific supports (Lane et al., 2025).

The purpose of the current study was to extend the findings of N. S. Pak et al. (2024) with additional Latino/a Spanish-speaking caregivers of autistic children while leveraging innovations such as a cyclical coaching approach to reduce weekly therapy time (Lane et al., 2016, 2025). Key differences between the N. S. Pak et al. (2024) study features and the current study are in Table 1. Some Latino Spanish-speaking families of autistic children who have participated in intervention studies have reported desiring more sessions or a longer intervention duration (e.g., McGuire et al., 2025; N. S. Pak et al., 2024). Thus, we adopted the cyclical coaching approach to add episodes of focused practice and coaching in each visit without reducing the overall duration of the intervention program in this iteration. We investigated the following research questions:

(1) Do Latina Spanish-speaking caregivers use EMT en Español strategies in interactions with their young children on the autism spectrum when strategies are taught using a lower-intensity, cyclical, systematic coaching approach? (primary dependent variable)
(2) Do children on the autism spectrum increase their frequency and diversity of communication when their caregivers have been taught to use EMT en Español strategies using a lower-intensity, cyclical, systematic coaching approach?
(3) Do caregivers find the approach to be socially valid, based on interview responses, survey responses, and study completion?

Based on findings from the earlier N. S. Pak et al. (2024) study, we hypothesized that caregivers would use EMT en Español Para Autismo when taught using this systematic coaching approach and that they would find the approach to be socially valid. Finally, we also hypothesized that caregivers would find the methods to be more manageable and would complete the study.

## 2. Materials and Methods

### 2.1. Participants

We recruited caregiver–child dyads who met the following criteria: (a) Spanish was the primary language spoken in the home, (b) the child was 24–47 months at the time of eligibility assessment, (c) the child had a diagnosis or demonstrated characteristics of an autism spectrum disorder (according to parent report and an administered autism screener), (d) the child demonstrated delayed language development (per standardized language assessment), (e) one primary caregiver was willing and able to participate in training during weekly sessions with their child for at least 5 months, and (f) the family lived locally or could otherwise attend visits in person. We recruited participants with support from local home visiting programs and autism support organizations. This study was approved by a University Institutional Review Board (IRB). Consent forms were developed and approved in English, then forward and backward translated by bilingual student research assistants. Discrepancies were resolved by consensus. Spanish consent forms were then approved by the IRB. Parents provided consent for themselves and permission for their children to participate. If children revoked assent by continuous crying or other signs of distress for at least 5 min, after which they were not able to be comforted by the caregiver, the visit ended for the day.

Three assessments were administered in Spanish or bilingually by the first author to determine eligibility. A study-specific caregiver survey gathered basic information related to the inclusion criteria (e.g., child age, caregiver willingness to participate). We administered the *Screening Tool for Autism in Toddlers and Young Children* (STAT; Stone & Ousley, 2008) to confirm the presence of autism characteristics. The STAT is an interactive screening tool for characteristics of autism related to play, motor imitation, and communication in children 24–36 months old (Stone & Ousley, 2008). We administered the *Preschool Language Scales, 5th edition Spanish* (PLS-5 Spanish; Zimmerman et al., 2012) to determine the presence of a language delay. Expressive Communication subscale scores on the PLS-5 Spanish are reported in Table 2. This subscale allows for more caregiver report and may be more valid than the Auditory Comprehension subscale, given children’s unclear engagement during several of the administered items.

Dyads who were eligible and enrolled in the study also completed a demographics survey, a language exposure survey, and two 20 min language samples (one in English and one in Spanish, approximately 1 week apart). Language sample data included the child’s number of different words and mean length of utterance in words in Spanish and English (see Table 2).

Our recruitment goal was five dyads. Five dyads were assessed for eligibility, four enrolled, and three completed the study. Dyad 4 did not complete the study due to numerous cancelations related to illnesses and other scheduling conflicts. Baseline characteristics for all dyads are reported in Table 2. Caregivers were all mothers. Their education levels varied from completing high school to completing a Bachelor’s degree. Using a conservative estimate of income to needs, all families were residing in low-income households with one family well below the federal poverty level. Children were 33–47 months old at enrollment. Children in Dyads 1, 2, and 3 had confirmed autism diagnoses; Child 4 was waiting for an evaluation, and he demonstrated autism characteristics on the STAT.

### 2.2. Design

The study was a multiple baseline design across sets of EMT en Español behaviors implemented by the caregiver. The design was replicated across dyads. Multiple baseline designs are single-case experimental designs in which the independent variable is introduced in a time-lagged fashion (Ledford & Tuck, 2024). This within-study replication of experimental effects is the basis for establishing internal validity in single-case experimental designs (Ledford & Ayres, 2024). Across design types in single-case experimental research, primary data are typically collected in the intervention context, and generalization data are collected as secondary dependent variables (Gast & Ledford, 2024).

In the current study, the independent variable was teaching caregivers to implement adapted EMT en Español strategies using a reduced intensity, cyclical approach to teach–model–coach–review. The primary dependent variable was caregiver use of EMT en Español strategies during coached caregiver–child interactions. Strategies were grouped based on child characteristics (see Section 2.4.1. Dependent Variable Measurement). Phase change decisions were made based on formative analysis and practical considerations for the participants. Secondary dependent variables included child communication and generalization of caregiver strategy use to uncoached interactions in contexts other than play.

### 2.3. Procedures

**Figure 1:**
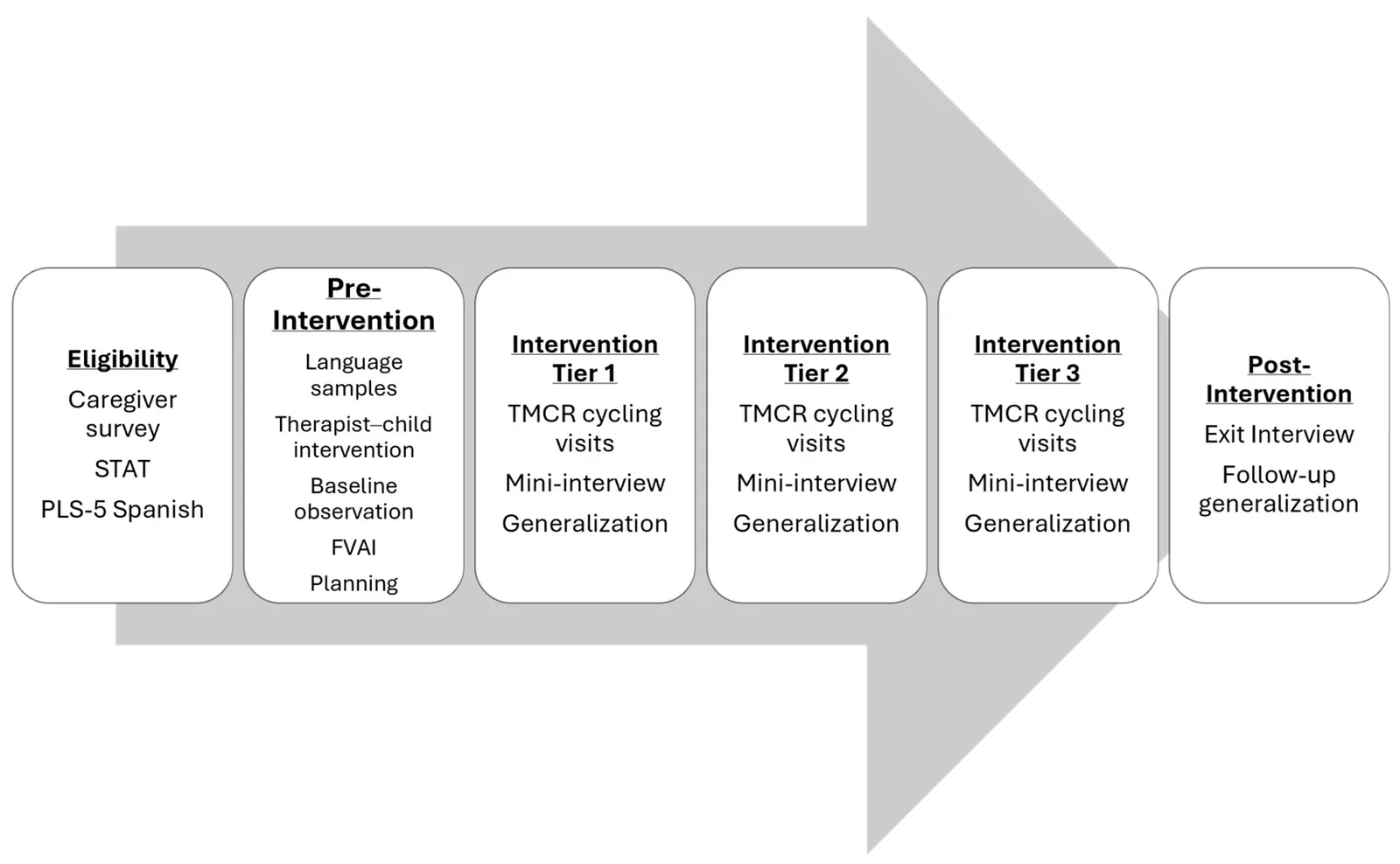
Study timeline for each dyad.

In the following sections, “visit” refers to a meeting with the dyad. “Session” refers to any of the various study activities that could occur during a visit. Visits took place primarily in person in family homes once per week. Figure 1 shows the timeline of activities for each dyad.

The therapist (first author) conducted all visits except for the post-intervention visit, which included the exit interview and follow-up generalization session. The therapist was female, multiracial (Korean and White), a native English speaker, and a Spanish speaker with 9 years of formal Spanish education. She had advanced Spanish conversational skills, including knowledge of autism terminology and intervention strategies. She was a licensed and certified speech language pathologist with 11 years of clinical and research experience working with autistic children and their families. A female bilingual undergraduate research assistant accompanied the therapist when possible to assist with recording and managing materials. This research assistant also conducted the follow-up visit. The research assistant was Latina, a native Spanish speaker, and a fluent English speaker.

Study activities were conducted in the caregiver’s preferred language, which was Spanish for Dyad 1 and Dyad 3. Dyad 2 spoke primarily Spanish at home, but both parents also spoke and understood English fluently. Thus, many study interactions occurred bilingually. Interactions with the child occurred in Spanish as much as possible to match the family’s typical home language environment and to allow parents to practice using target-level language in Spanish. All written materials were provided in Spanish.

#### 2.3.1. Baseline

Six 5 min baseline sessions were conducted across six visits with each dyad. The therapist instructed the caregivers to play with their children as they normally would. The therapist made a set of toys available for the families to play with (blocks, stamps, colored pencils and paper, baby dolls, tea and cookies set, book, puppet), but they were not required to play with specific toys. The therapist did not provide any teaching, modeling, coaching, or reviewing during baseline visits.

#### 2.3.2. Individualization

Collaboration with families is a key feature of EMT en Español Para Autismo intervention (N. S. Pak et al., 2024). The heterogeneity in the needs and preferences of autistic children as well as the cultural and linguistic heterogeneity of Latino families in the United States warrants several methods of individualization (L. Cycyk & Iglesias, 2015).

Four 20 min therapist–child intervention sessions were implemented directly with the child to provide additional intervention dosage and informal data for individualization. Therapist–child intervention sessions occurred immediately after baseline sessions at four visits. Caregivers were told they were welcome but not required to observe. No caregiver instruction was provided. The therapist implemented all EMT en Español target strategies with the child. Although there was a risk that caregivers would learn strategies from observation alone which would threaten internal validity, this did not occur in prior studies (N. S. Pak et al., 2024). The therapist took notes on the child’s behavior and communication in a Visit Log completed in REDCap (Harris et al., 2019) after each visit. For example, the therapist noted the child’s preferred toys, response to strategies, and joint engagement. The therapist also reviewed videos of at least two sessions for each dyad and completed a Child Observation Protocol (Supplementary File S1) to structure planning for intervention.

One Family Values and Activities Interview (FVAI; Peredo, 2016) was administered to the caregiver. This approximately 60 min semi-structured ethnographic interview asks caregivers about their family values, support system, and child communication contexts. The FVAI protocol is designed to build therapist–family rapport, facilitate toy selection, and provide information for generalization planning and measurement.

One 30 min planning session was conducted with each family immediately prior to intervention. The families collaborated with the therapist to select 3–4 toy sets (approximately $50) to keep and use in intervention (e.g., puppets, magnetic blocks, animal shape sorters). During the planning session, an AAC device and initial training were offered to each dyad regardless of the child’s spoken language abilities, as many autistic individuals use both speech and AAC (Koerner et al., 2023). An Apple iPad (10.9-inch, 10th generation; Apple Inc. 2025, Cupertino, CA, USA) with a communication app TDSnap (version 1.36; Tobii Dynavox LLC, 2025) was selected due to its relatively low cost and straightforward toggling between Spanish and English. Dyad 1 used TDSnap at the beginning of the study but decided to stop using it before intervention session 10. Dyad 2 used Proloquo2Go (version 8.6; AssistiveWare, 2025) consistently throughout the study, which they had already purchased and begun programming independently before enrolling. Spanish and English page sets were available on the apps, and the therapist helped the families program activity pages with vocabulary corresponding to study activities, family routines, and child interests (Beukelman & Light, 2020). Dyad 3 chose not to use AAC.

#### 2.3.3. Intervention

**Figure 2:**
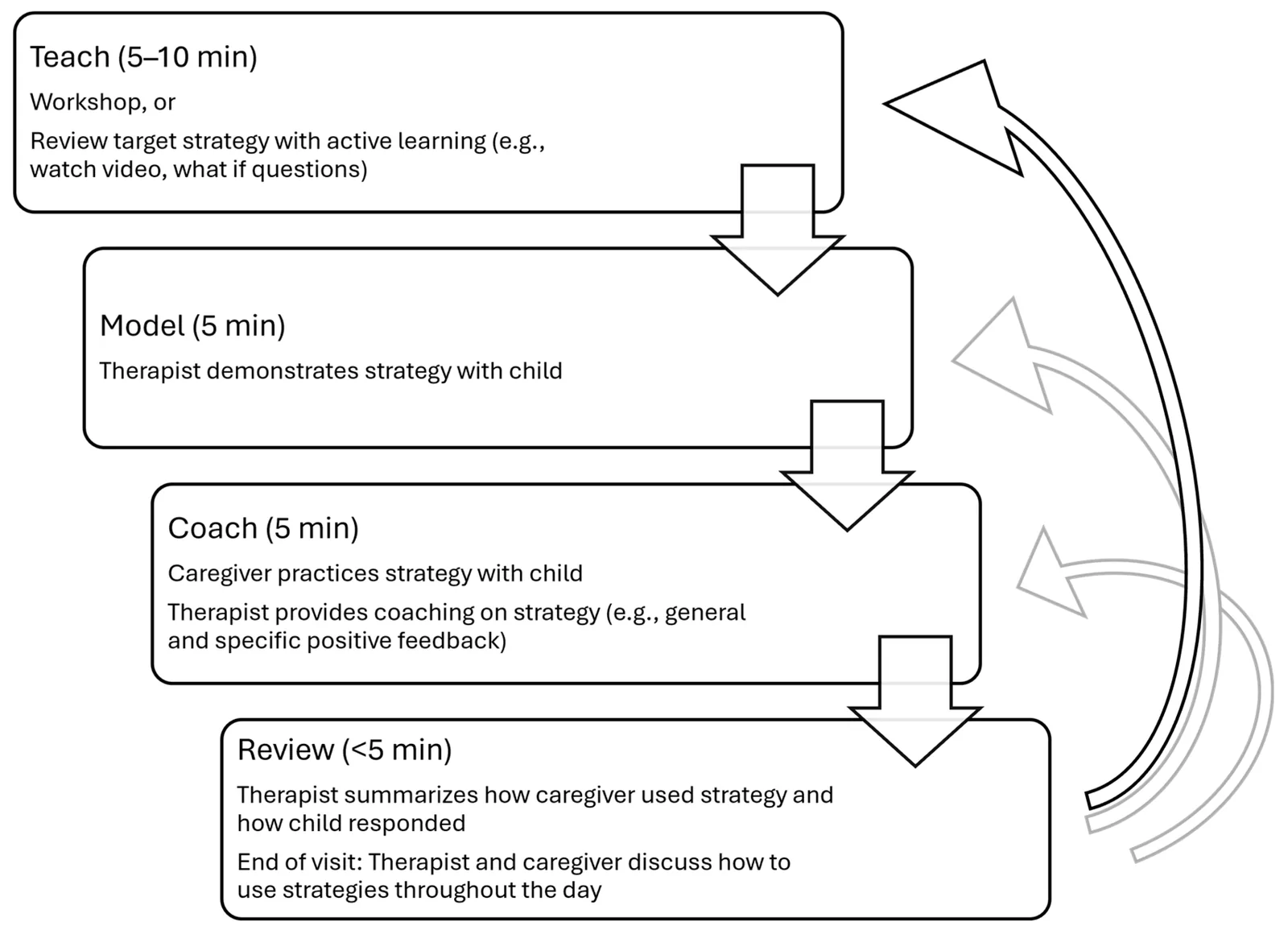
Intervention visit structure.

Caregivers were taught using teach–model–coach–review (TMCR; Roberts et al., 2014) during intervention sessions. Figure 2 shows the cyclical TMCR procedures. The first TMCR session of each tier began with a workshop to introduce the strategy set. Subsequent TMCR cycles followed the same structure described by N. S. Pak et al. (2024); however, each segment was shortened, multiple TMCR cycles were conducted per visit (Lane et al., 2025), and overall intensity was reduced. Typically, one or two full cycles were completed in a visit. After the review segment, all or part of the TMCR cycle was repeated, depending on time, caregiver preference, and child engagement. The 5 min “coach” portion of each cycle was coded for primary data; therefore, there were up to two datapoints per visit.

#### 2.3.4. Generalization and Maintenance

Up to four 5 min generalization sessions were conducted (M = 2, range 0–4). The purpose of these activities was to observe whether caregivers generalized use of the EMT en Español strategies to activities other than play without coaching. The therapist asked the caregiver to perform a household routine (e.g., brushing teeth, snack, reading a book) as they typically would. Information from mini-interviews and informal conversations with the family were used to select a generalization context. Dyad 2 typically selected a reading context, Dyad 3 selected brushing teeth, and Dyad 4 selected snack time. Generalization sessions were not conducted with Dyad 1 due to scheduling conflicts. Generalization sessions occurred up to one time during each tier of intervention.

One 60 min exit interview was conducted by a bilingual research assistant 3–5 weeks post-intervention, along with a follow-up generalization session.

### 2.4. Measures and Data Collection

Measures are similar to those used in the study by N. S. Pak et al. (2024). Eligibility assessments are described in Section 2.1.

#### 2.4.1. Dependent Variable Measurement

The caregiver’s frequency of use of target EMT en Español strategies during 5 min caregiver–child interactions was the primary dependent variable. During baseline, sessions were not coached; during intervention, these were all 5 min coaching sessions that occurred in the visit. Coding definitions were based on those used in prior research (N. S. Pak et al., 2024; N. Pak et al., 2025).

For children who used spoken language (Dyads 1 and 3), the following three sets of EMT en Español strategies were targeted: (a) contingent modeling of target-level and proximal target-level language, (b) linguistic expansions, and (c) time delays. For children who used minimal spoken language (i.e., 0–5 different words, see Table 2, Dyad 2), the following three sets of strategies were targeted: (a) contingent modeling of target-level language, (b) contingent modeling of proximal target-level language, and (c) time delays. For all caregivers, Spanish was the target language for intervention; however, targets or proximal targets in Spanish or English were coded.

Utterances were coded as contingent when they were (a) in response to a child turn and related to what the child communicated or (b) after a 3 s pause and related to the shared activity. Language targets were selected for children based on the child’s spoken language at baseline (Kaiser & Pak, in press; N. S. Pak et al., 2024; N. Pak et al., 2025). All children used fewer than 50 different words in Spanish and English (see Table 2) and their mean length of utterance was less than 1.5 words. Target language utterances were short grammatical phrases typically including one content word (e.g., article + noun, inflected verb). Proximal target language utterances were slightly more complex, typically containing two or three content words (e.g., inflected verb + object). In Tier 1, caregivers were also taught to use aided AAC modeling, or activating words using the communication app while they spoke language targets aloud (Beukelman & Light, 2020). Language targets in any mode were coded (AAC, speech, or both).

For time delays, we taught caregivers to select moments when the child was engaged in an activity, present a choice-making opportunity by holding up two objects and pausing expectantly, and respond to child communication (e.g., reaching toward an object) with target-level language.

The child’s communicative acts were measured during the same 5 min coaching sessions. Child language variables were number of total words, number of different words, and number of communicative acts (gestures, words, and potentially communicative vocalizations). For vocalization to be considered potentially communicative, a secondary indicator of communicative intent needed to be present, such as a vocalization paired with a gaze shift or temporal contingency to the caregiver’s play action. Words were counted regardless of language (Spanish, English) and mode (speech, AAC, or both).

#### 2.4.2. Social Validity Procedures

Up to three 10–15 min mini-interviews were conducted during the intervention phase to formatively assess caregivers’ satisfaction with and impressions of the effects of the ongoing intervention. Questions included “How does the program feel to you?”, “What has changed about the activities your family regularly participates in?”, “How do you typically communicate with your child?”, and others. These were conducted at the beginning or end of a weekly visit to minimize additional visits.

An exit interview provided quantitative and qualitative data on caregiver perceptions of the intervention. Open-ended questions included questions related to how often caregivers used the strategies throughout the week and during what activities, whether they taught the strategies to other primary caregivers, their confidence implementing the strategies, and what they found most and least helpful about the intervention. A series of additional questions asked caregivers to rate how effective and appropriate they found each of the discrete strategies, how effective and appropriate other Latino families would see the discrete strategies, and how well they fit with their views of interacting their children. For example, caregivers were asked during the exit interview about the strategy *observar y responder* (notice and respond), which was taught as a component of contingent language modeling. Caregivers responded on a scale from 1 (ineffective and inappropriate) to 5 (very effective and appropriate). They were encouraged to provide rationale for their responses.

#### 2.4.3. Data Collection, Fidelity, and Interobserver Reliability

The primary coders were three bilingual undergraduate students majoring in fields related to speech language pathology, sign language interpreting, or medicine. They were all native Spanish speakers. All coding was completed from video as soon as possible after a session using SALT 24 software (Miller & Nockerts, 2024) and the study coding manual (adapted from N. Pak et al., 2025). Coders did not attend visits and were not informed when phase changes occurred; however, they could not be kept completely naïve to study phase because of the obvious presence or absence of coaching in the videos. Coders were trained on bilingual Spanish–English SALT transcription using free modules on the SALT website. Prior to coding data from the study, they coded practice videos and received feedback from the first author. When the study began, the first author and coders double-coded each observation until they reached at least 80% reliability. Then the coder reviewed and corrected all previously coded transcripts.

Following training, one-third (33%) of sessions (35 sessions), distributed across study phases, were randomly selected and coded by a second coder (the first author or another undergraduate coder). Codes were compared to obtain reliability percentages. Average reliability was 86% (55–95%) for overall coding, 80% (41–94%) for child codes, and 88% (59–96%) for caregiver codes. The team met weekly throughout the study to compare codes, discuss discrepancies, and update the coding manual to clarify rules. Consensus codes were used as primary data when overall codes or caregiver codes (the primary dependent variable) were below 80% for an observation. This occurred for three sessions.

Procedural fidelity was measured for 100% of baseline and intervention visits (with at least one data collection session) by the same undergraduate research assistant who assisted on visits. The research assistant watched videos of visits and completed fidelity checklists on REDCap (Harris et al., 2019). Average fidelity was 98% (80–100%) for baseline observations and 97% (76–100%) for TMCR visits. In some cases, low fidelity was due to intentional modifications to the procedures to support participation. For example, if child engagement seemed fragile (e.g., beginning to wander away from the play area), we sometimes abbreviated the teaching segment and omitted the modeling segment, given that families in prior studies reported coaching to be the most valuable component of TMCR (N. S. Pak et al., 2024; Peredo et al., 2022).

### 2.5. Data Analysis

Caregiver behaviors were graphed using GraphPad Prism 11 for Windows version 11.0.2 (GraphPad Software, LLC, 2026) and scaled to 3:2 to improve reliability (Radley et al., 2018). Per single-case design standards, we used visual analysis during the study to support phase change decisions (formative analysis) and at the end of the study to judge the presence or absence of functional relations (summative analysis). In time-lagged single-case designs, formative analysis typically involves both within-tier and across-tier comparisons (Ledford & Lambert, 2024). In other words, intervention is introduced on a given tier (a set of strategies, in this case) when data are sufficiently stable in baseline and in the other tiers. These response-guided decisions were made when possible; however, there were occasions when practical reasons dictated phase changes. For example, Dyad 1 requested around session 17 that the remaining number of visits be limited to about a month due to new constraints on their schedule. Thus, Tier 2 strategies were introduced, continued for four sessions (two weekly visits), and then Tier 3 strategies were introduced and continued for four sessions (two weekly visits).

At the end of the study, we determined a functional relation between the dependent variable and the independent variable was present if (a) the introduction of TMCR in each tier corresponded with more frequent caregiver use of the strategy and (b) the changes occurred in all three tiers (Ledford & Lambert, 2024). All authors had expertise in single-case experimental design, and visual analysis judgments were made by consensus.

In addition to visual analysis, Tau-U was calculated to estimate effect sizes using the Single-Case Effect Size Calculator “Tau” effect size index (Pustejovsky et al., 2024). Tau-U is a nonoverlap statistic used to make pairwise comparisons between baseline and treatment observations (Parker et al., 2011). In other words, Tau-U can reflect intervention effects above and beyond baseline level and trend. Although effect sizes were calculated to support visual analysis and interpretation, they are not meant to stand alone and are discussed in the context of the desired change and practical significance (Vannest & Ninci, 2015). Tau estimates are fairly robust to small sample size, non-normal data, and outliers, resulting in more stable sampling distributions (Ferron et al., 2023). Unlike some effect size indices, Tau is interpretable even with zero baselines, which we anticipated for some behaviors based on prior studies (N. S. Pak et al., 2024; Peredo et al., 2018). Although nonoverlap indices help quantify a change in the desired direction between phases, ceiling effects may limit the degree to which the effect sizes vary. Further, a correction can be used (Tau-BC) to control for baseline trend prior to calculating effect size. To ensure consistent analyses across participants, baseline corrected Tau (Tau-BC) was applied regardless of baseline trend significance (Ferron et al., 2023).

## 3. Results

### 3.1. Attendance and Attrition

The three dyads who completed the study participated in 25–33 visits, including two to three visits for eligibility assessments, six baseline phase visits, 11–14 TMCR visits, and one follow-up visit. Remaining visits were generalization sessions, interviews, planning sessions, or workshops. Although study activities generally followed the same sequence across participants, the number of sessions in a visit was flexible. The total coaching time ranged from 95 to 125 min with a mean of 107 min. The duration of participation from the first baseline session to the last intervention session was 5–10 months, including breaks such as the winter holidays. Dyad 4 completed 15 visits, including two visits for eligibility assessments, six visits with baseline sessions, and six visits with TMCR (30 min of total coaching time). Baseline and intervention visits spanned 6 months.

### 3.2. Caregiver Strategy Use

**Figure 3:**
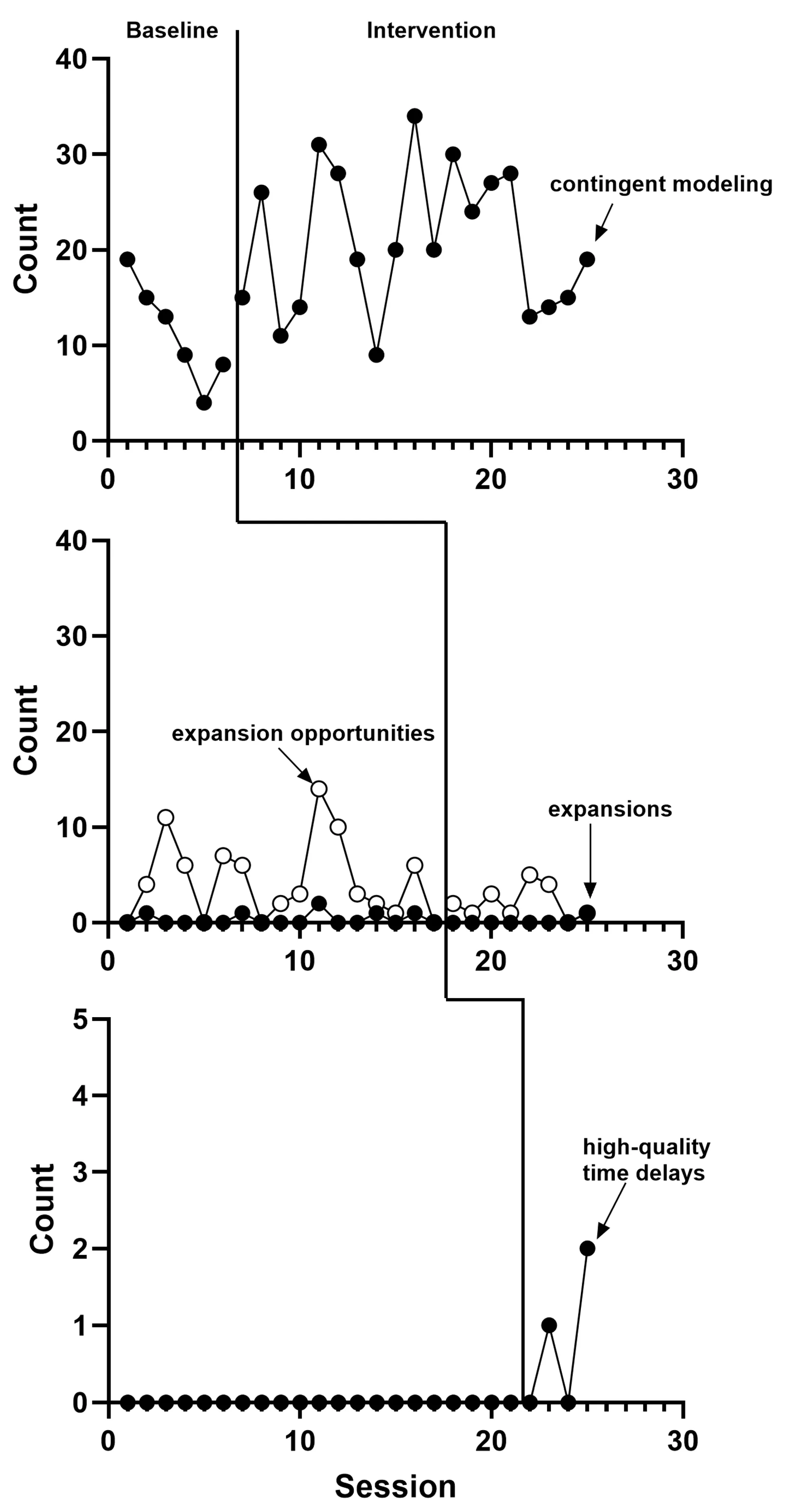
Caregiver data for Dyad 1.

Caregiver 1 was taught contingent modeling of targets and proximal targets in Tier 1, expansions in Tier 2, and time delays in Tier 3 (see Figure 3). In Tier 1, baseline data followed a decreasing trend and there was an immediate increase in level after beginning intervention (M = 11.3, range 4–19). Variability was high during the TMCR condition and intervention data substantially overlapped with baseline (M = 20.9, range 9–34). For Tier 2, baseline data were low and stable (M = 0.35, range 0–2). No change was observed when expansions were taught during the TMCR instruction (M = 0.13, range 0–1). Tier 3 data for time delays also were at zero levels during baseline. Caregiver 1 immediately increased her use of time delays when TMCR was introduced; however, data overlapped with baseline (i.e., observations in which she used no time delays still occurred; M = 0.75, range 0–2). Overall, effects of cyclical TMCR instruction on Caregiver 1’s use of EMT en Español strategies were not consistent across tiers; therefore, no functional relation was demonstrated. No generalization data for Dyad 1 were collected due to scheduling conflicts.

Effect sizes reported in Table 3 are consistent with visual analysis. For Dyad 1, the calculated effect size indicates a large effect in Tier 1 (Tau-U_BC_ = 1.00), reflecting an increase in contingent modeling with coaching. There was no effect evident in Tier 2 (Tau-U_BC_ = −0.18), consistent with minimal use of expansions evident in Figure 3. The effect size estimate for Tier 3 was moderate (Tau-U_BC_ = 0.50), reflecting the slight increase in caregiver use of time delays seen in Figure 3.

**Figure 4:**
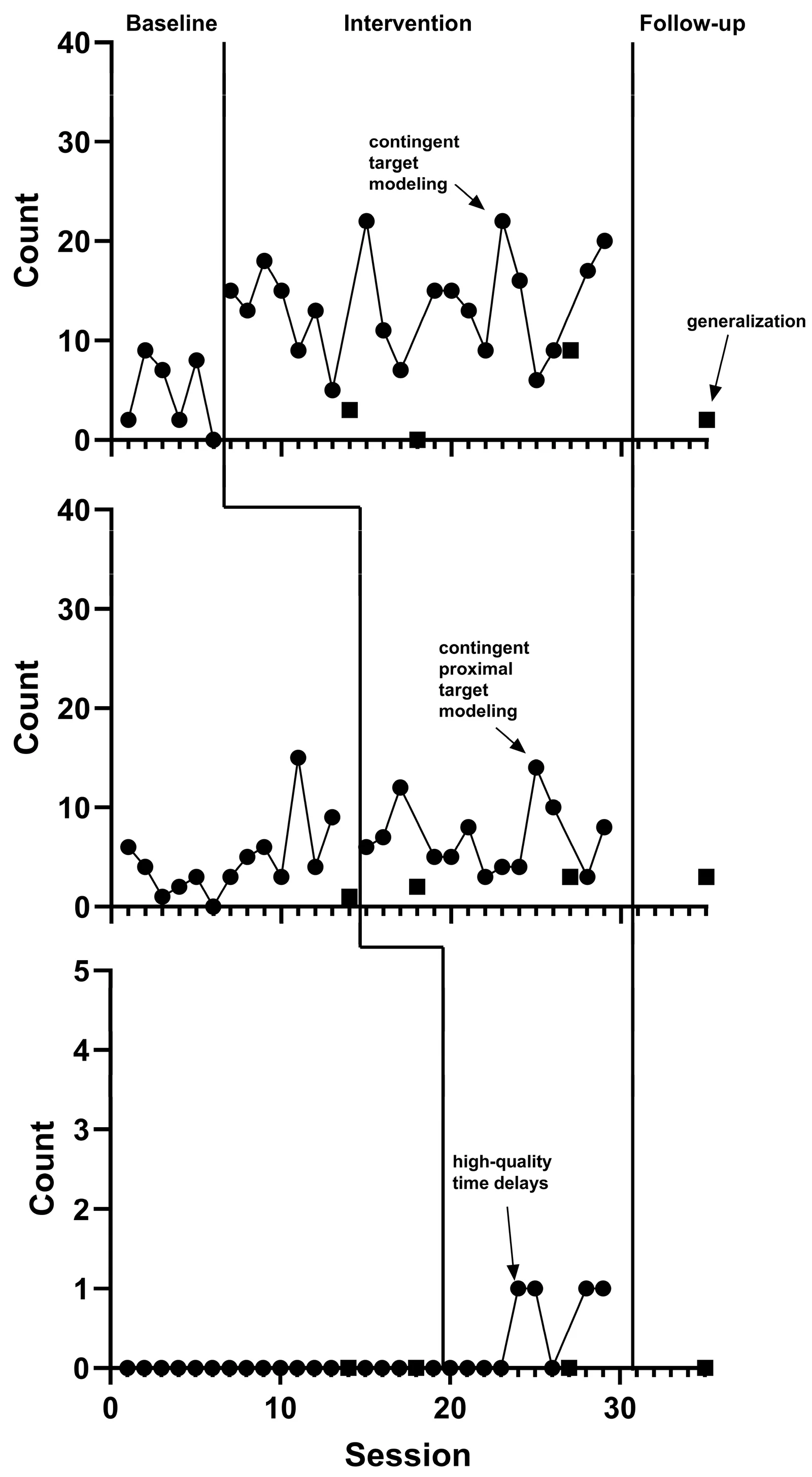
Caregiver data for Dyad 2.

Caregiver 2 was taught contingent modeling of specific target-level language in Tier 1, contingent modeling of proximal target-level language in Tier 2, and time delays in Tier 3 (see Figure 4). Data were low and variable for specific target-level language in baseline (M = 4.7, range 0–9) and immediately increased in level when intervention was introduced (M = 13.5, range 5–22). Data remained highly variable throughout the intervention phase and overlapped with baseline. In Tier 2, baseline data (M = 4.7, range 0–15) began to increase when Tier 1 intervention was introduced from a mean of 2.7 proximal targets per 5 min session (range 0–6) to a mean of 6.4 (range 3–15). There were no clear changes in level, trend, or variability of proximal target use when Tier 2 TMCR intervention was introduced, based on visual analysis (M = 6.8, range 3–14). The use of time delays (Tier 3) remained at zero during baseline. Caregiver 2 immediately attempted a time delay when taught this strategy in the first intervention session but correct use of time delays did not increase until the fifth session of Tier 3 intervention. The use of EMT en Español strategies in generalization contexts (book-reading) remained at baseline levels throughout the intervention phase in all tiers.

For Dyad 2, effect sizes indicated a large effect for Tier 1 (Tau-U_BC_ = 1.00), consistent with the increase in caregiver use of contingent target modeling evident in Figure 4. Given that contingent proximal target modeling increased in the baseline phase in Tier 2, the effect size was moderate and negative (Tau-U_BC_ = −0.40), reflecting a slight decrease in the use of proximal targets after intervention was introduced. The effect size estimate was moderate in Tier 3 (Tau-U_BC_ = 0.44), indicating a slight increase in the use of time delays after coaching.

**Figure 5:**
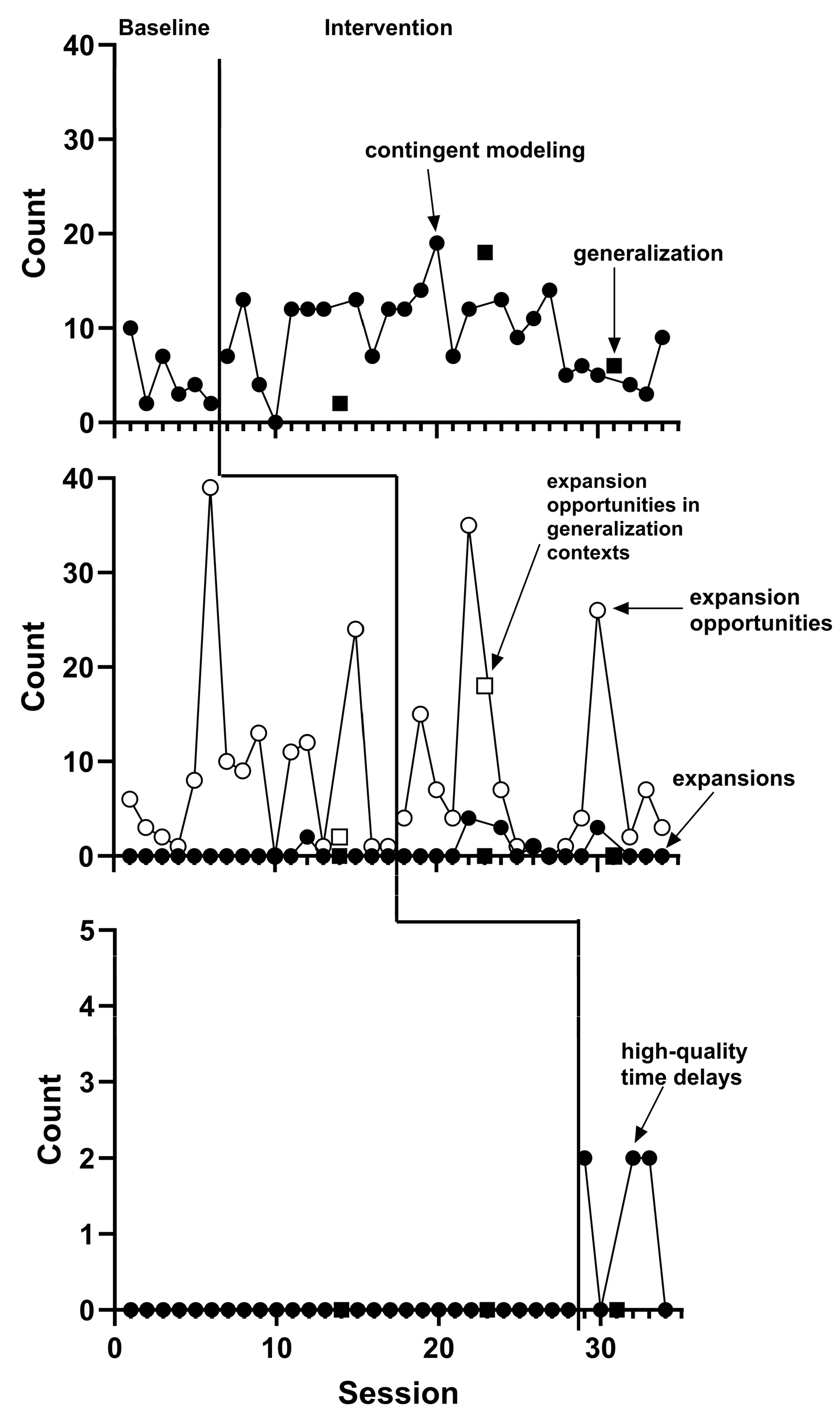
Caregiver data for Dyad 3.

Caregiver 3 was taught contingent modeling of targets and proximal targets in Tier 1, expansions in Tier 2, and time delays in Tier 3 (see Figure 5). The use of target language decreased across baseline observations (M = 4.7, range 2–10). Targets immediately increased when the intervention was introduced; however, data were variable, with significant overlap with baseline (M = 9.4, range 0–19). The use of contingent modeling further decreased later in the intervention phase, when time delays were introduced in Tier 3. The use of expansions (Tier 2) and time delays (Tier 3) were low and stable during baseline (M = 0.1 and M = 0, respectively). There was a small increase in the use of expansions in the fifth session after introducing TMCR in Tier 2; however, the number of expansions in this session (4) remained low relative to the number of child-presented opportunities to expand (35). Data subsequently returned to baseline levels (overall M = 0.7, range 0–4). When time delays were taught in Tier 3, Caregiver 3 immediately attempted several time delays in each session, although data during the intervention were variable and overlapped with baseline. Caregiver 3 used 0–2 high-quality time delays (M = 0.75) in each 5 min session during intervention. Generalization data (the use of strategies during brushing teeth routine) remained at baseline levels. Effect sizes for Dyad 3 indicate a strong effect in treatment Tier 1 (contingent target modeling; Tau-U_BC_ = 1.00) and a moderate effect in Tier 3 (time delays; Tau-U_BC_ = 0.60). There was no effect evident for Tier 2 expansions (Tau-U_BC_ = 0.21).

**Figure 6:**
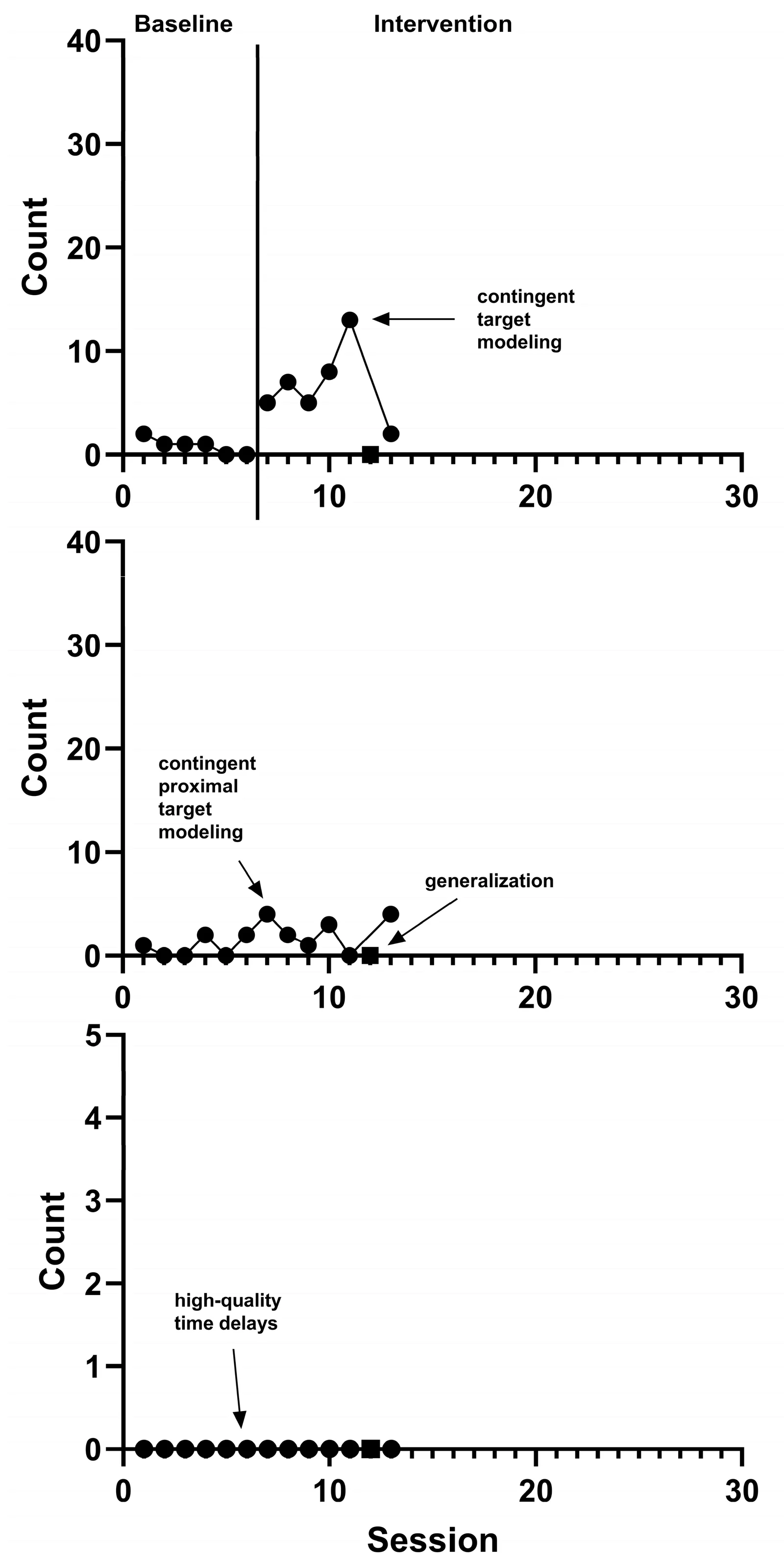
Caregiver data for Dyad 4.

Dyad 4 was taught contingent modeling of target-level language in Tier 1 and would have been taught contingent modeling of proximal target-level language and time delays in subsequent tiers (Figure 6). Baseline data were low and stable across EMT en Español strategies. When Tier 1 strategies were introduced, there was an immediate increase in level. In the first five sessions, data did not overlap with baseline. The sixth intervention session occurred after a long break (6 weeks) and data returned to baseline levels. One generalization session was conducted during a snack routine and data for all EMT en Español strategies were at baseline levels. Effect size for Dyad 4’s Tier 1, contingent target modeling, indicated a large effect (Tau-U_BC_ = 1.00).

### 3.3. Child Communication

**Figure 7:**
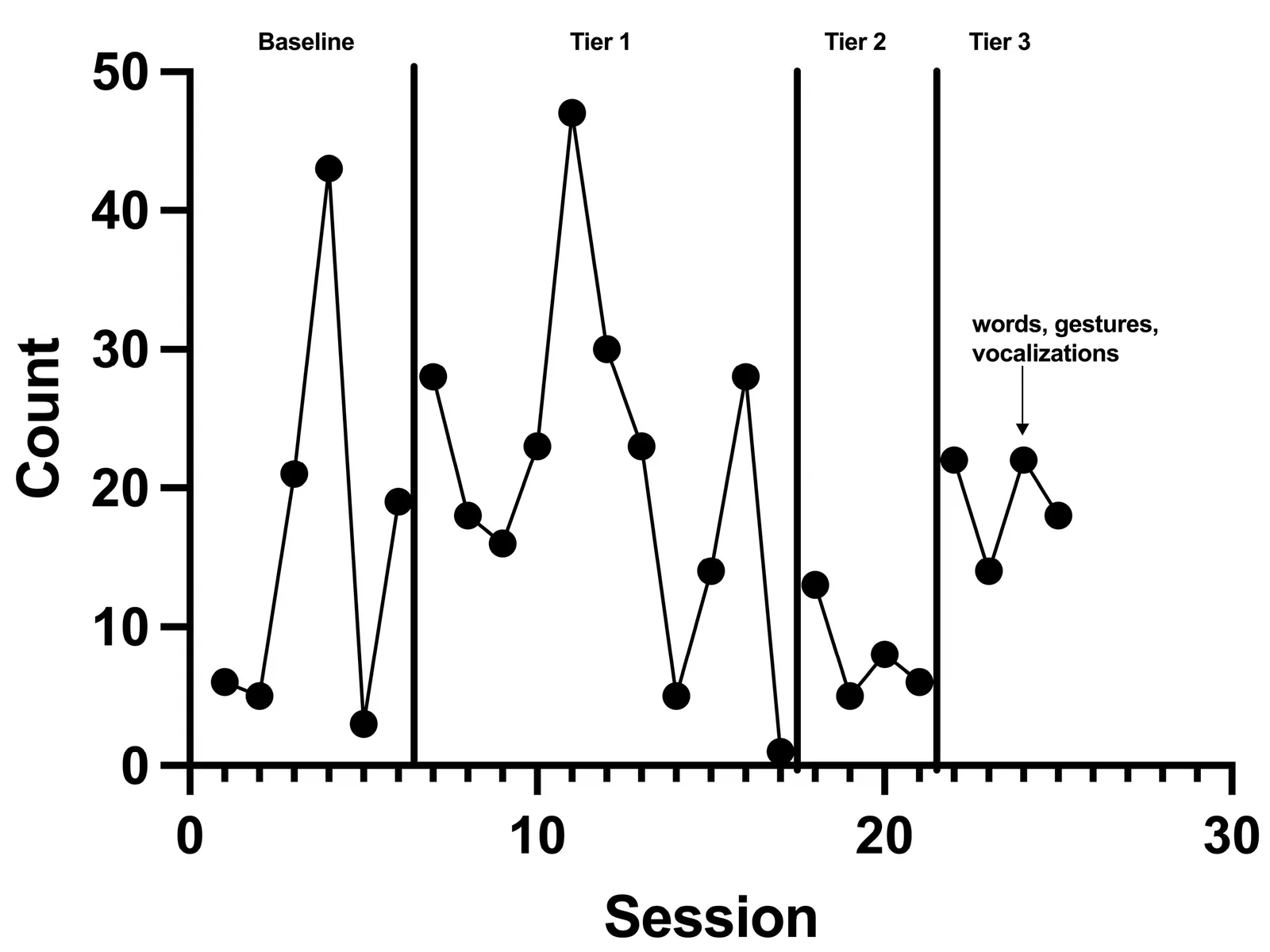
Child data for Dyad 1.

**Figure 8:**
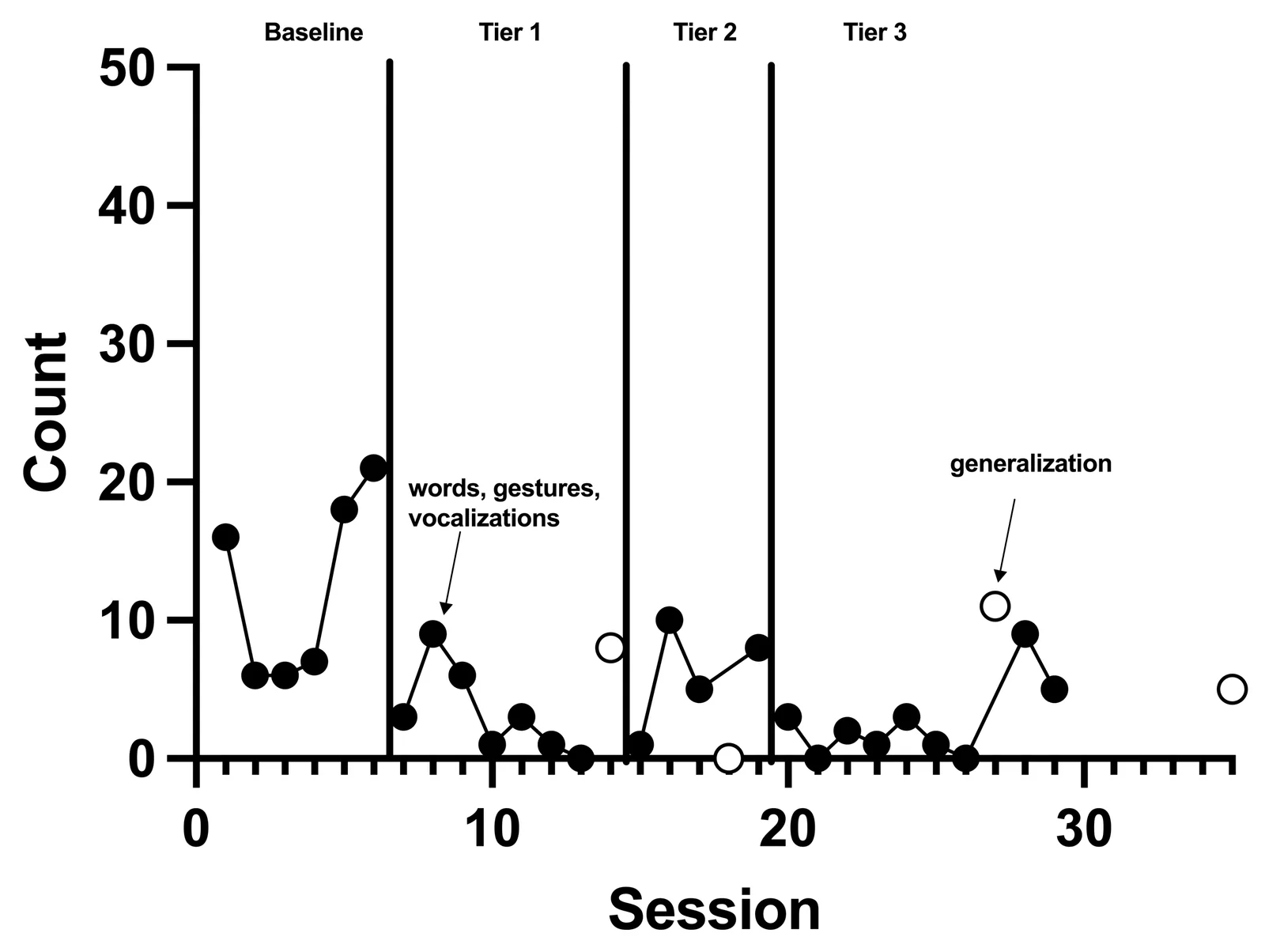
Child data for Dyad 2.

**Figure 9:**
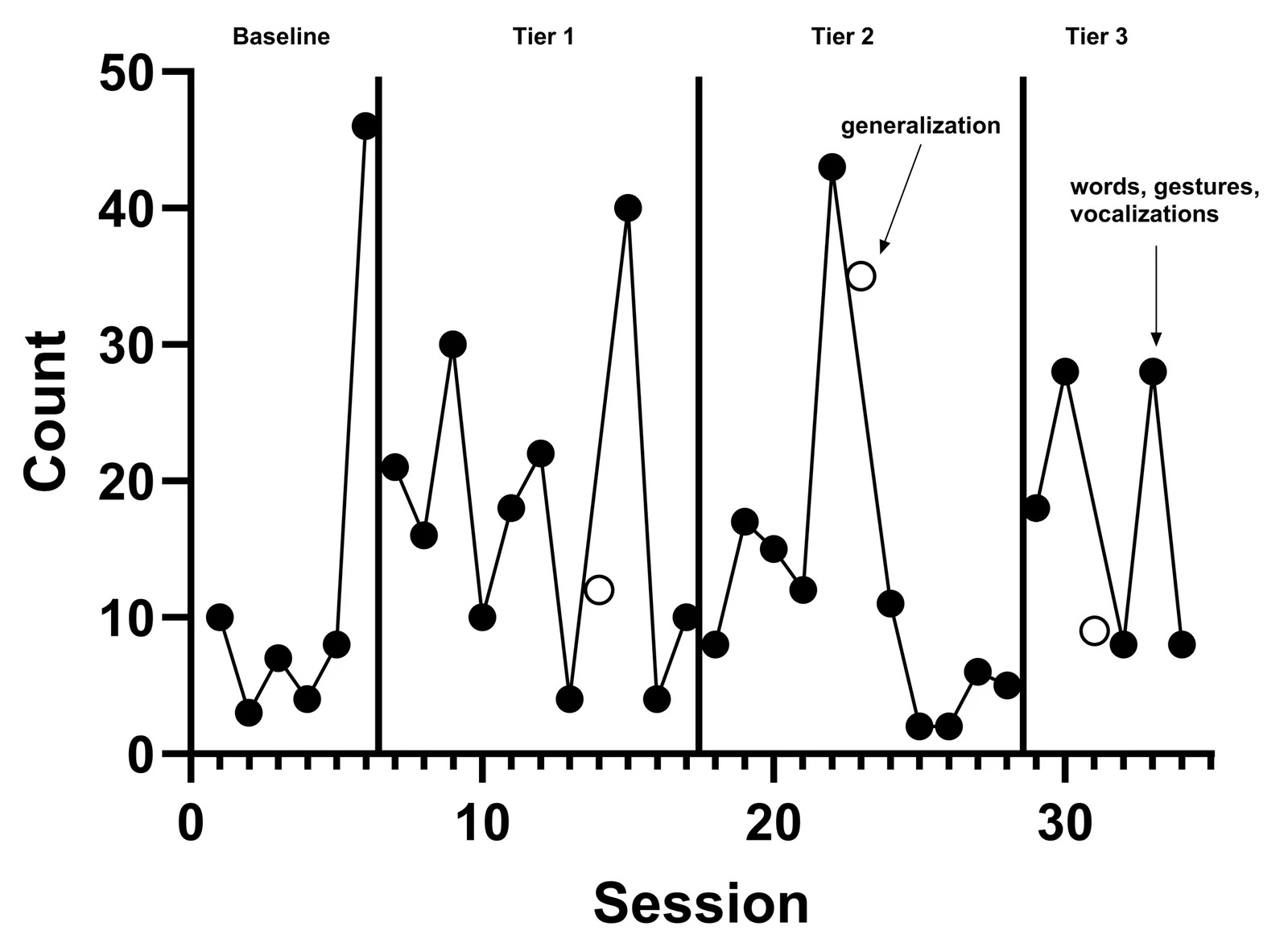
Child data for Dyad 3.

**Figure 10:**
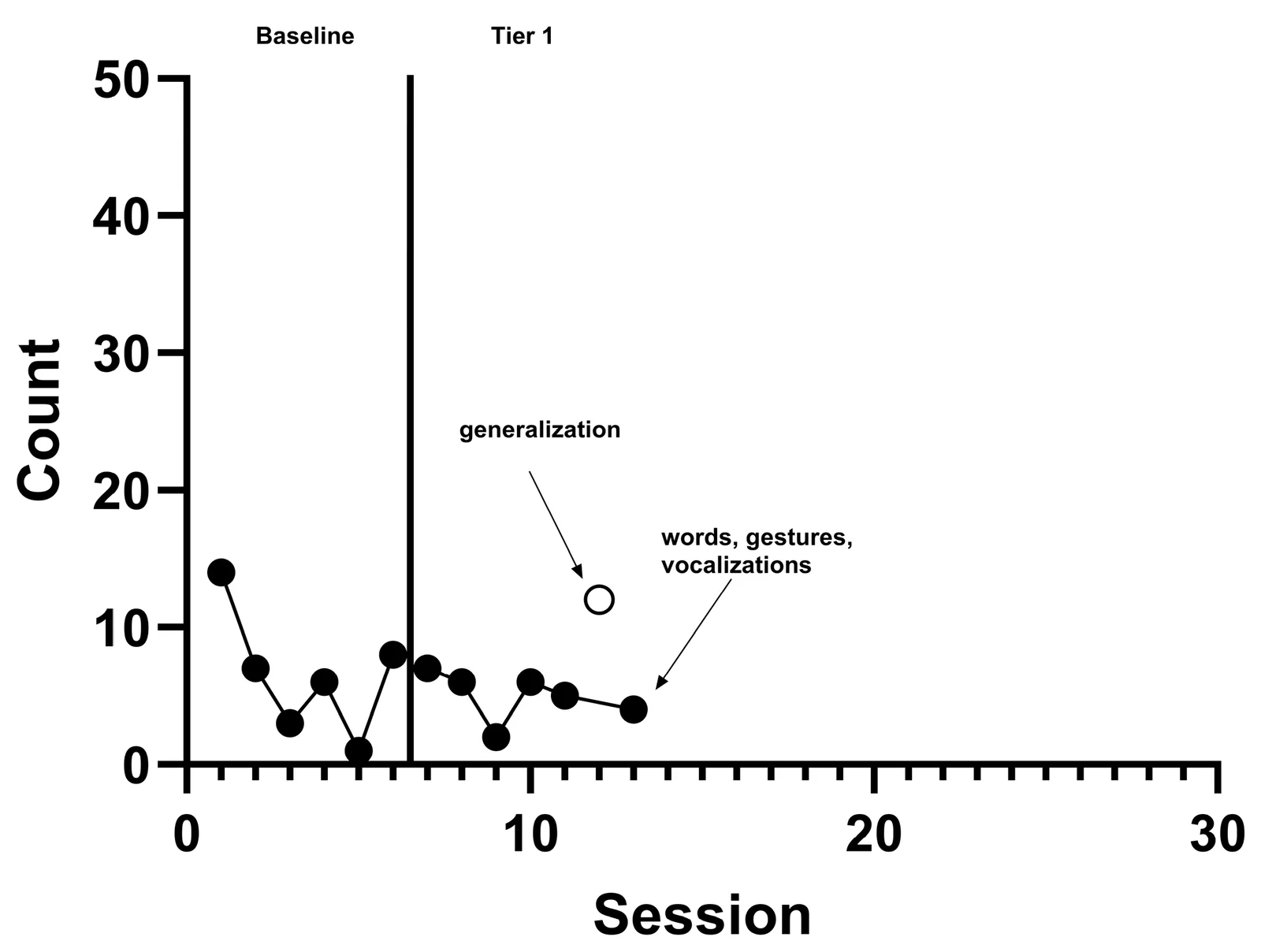
Child data for Dyad 4.

To aid the interpretability of the graphs, children’s number of communicative acts are displayed in Figure 7, Figure 8, Figure 9 and Figure 10, while data on number of total words and number of different words are in Supplementary File S3 in Supplementary Materials.

Child 1’s numbers of communicative acts per session are shown in Figure 7. In baseline, Child 1 used 0–14 total words (M = 4.7), 0–8 different words (M = 3.2), and 3–43 communication acts (words, gestures, or communicative vocalizations; M = 16.2) per 5 min session. Child 1’s communication acts remained highly variable with a decreasing trend throughout Tier 1 and Tier 2 intervention. Communication acts (mostly in the form of vocalizations and gestures) then increased immediately from an average of 8 in Tier 2 (range 5–13) to an average of 19 in Tier 3 (range 14–22) after his caregiver was taught time delays.

Child 2’s communication data are shown in Figure 8. In baseline, Child 2 used 0–5 total words (M = 1.7), 0–4 different words (M = 1.5), and 6–21 communication acts (M = 12.3) per 5 min session. Child 2 often used gestures such as placing his mother’s hand on objects to make requests for help, especially during certain activities (e.g., playing with pop beads, session 6). He continued to use very few words (0–3) per session throughout the study. AAC was available and within reach during sessions, but he did not use it communicatively during the study. Child 2’s data were influenced by his engagement and sensory needs throughout the study. For example, he was more engaged during after-school sessions when he wore noise-canceling headphones to school.

Child 3’s communication data are shown in Figure 9. In baseline, Child 3 used 1–43 total words (M = 12.0), 1–33 different words (M = 8.8), and 3–46 communication acts (M = 13.0) per 5 min session. Communication rate variability appeared to be influenced by the activity selected. Although we encouraged play with toys, Caregiver 3 sometimes selected pre-academic activities such as flashcards, puzzles, or storybooks, which led to outliers in Child 3’s data. For example, in Session 6 with flashcards, Child 3 named many pictures when prompted, resulting in an unusually high total word count of 43.

Child 4’s communication data are shown in Figure 10. In baseline, Child 4 used 0–1 total words (M = 0.7), 0–1 different words (M = 0.7), and 1–14 communication acts (M = 6.5) per 5 min session. Data were similar in Tier 1 intervention, with 0–2 total words (M = 0.5), 0–2 different words (M = 0.5), and 2–7 communication acts (M = 5.0).

### 3.4. Social Validity

Dyads 1, 2, and 3 completed exit interviews. All three caregivers reported that the “model” segment of the TMCR cycles was most helpful. Dyad 2 reported that the coaching component was helpful; Dyad 3, however, found coaching to be the least effective component. All three caregivers expressed confidence in implementing the strategies and reported using them throughout the week (approximately 2 h per week for Dyads 1 and 3 and 3.5 h per week for Dyad 2). Two caregivers taught strategies to other caregivers (husband, child’s teacher). Caregiver 1 felt that her child had more vocabulary due to the intervention, and she expressed appreciation that her child’s interests were incorporated into the play observations. Caregiver 2 reported that the intervention helped her understand how to use AAC and that she had seen improvement in her child. Caregiver 3 suggested that the intervention would be better with more time to play with the child. It was unclear whether she felt more modeling, more coaching, or more time in both would have been helpful.

Across all three caregivers, the strategies they found to be most effective and appropriate were contingently modeling target language (Tier 1) and using time delays (Tier 3), which aligns with effects observed during the intervention sessions. *Toma de turnos* (balanced turn-taking) and *no hacer tantas preguntas* (asking fewer questions) were strategies taught in Tier 1 as a component of contingent modeling. Caregivers rated these as effective and appropriate, but Caregivers 1 and 3 rated them slightly lower when asked whether other Latino families would find them effective and appropriate. The perception of the strategy of limiting instructions varied across caregivers: Caregiver 1 rated it as somewhat effective and appropriate, Caregiver 2 as very effective and appropriate, and Caregiver 3 as ineffective and inappropriate.

## 4. Discussion

The aim of this study was to test the effects of a lower-intensity, cyclical teach–model–coach–review approach on caregiver use of EMT en Español Para Autismo strategies with their autistic toddlers during play. Overall, there were demonstrations of effects for Tier 1 strategies (contingent modeling) with moderate to strong effect sizes and Tier 3 strategies (time delays) with low to moderate effect sizes. Tier 2 strategies (the use of expansions or contingent modeling of proximal targets) did not increase for these dyads when introduced. Thus, while there is some evidence across dyads that the independent variable influenced certain behaviors for these caregivers, there were no functional relations for any individual design. The overall effects on caregiver and child behavior were small.

The cyclical TMCR approach (Lane et al., 2025) and reduced overall intensity were the key differences from the N. S. Pak et al. (2024) study. The duration of the study for each dyad was 5 months or more, similar to previous research on EMT en Español Para Autismo. However, the weekly number of visits was reduced to one. While weekly visits for several months is still a considerable time commitment for families with young children with disabilities, it was significantly reduced from the visit frequency in the N. S. Pak et al. (2024) EMT en Español Para Autismo study (up to three times per week), far fewer than what is recommended in intensive ABA interventions for many autistic children, and comparable to weekly visit frequencies proposed in other NDBI studies considered low-intensity (Lane et al., 2025; Rollins et al., 2021).

The time for coaching in each weekly visit was reduced to 5–10 min, whereas coaching in prior EMT en Español studies has been 15 min or more, multiple times per week (Bailey et al., 2024; N. S. Pak et al., 2024; Peredo et al., 2018; Peredo et al., 2022). Overall intensity of coaching time in the N. S. Pak et al. (2024) study was over 400 min per dyad, while it was 125 min or less in the current study (see Table 1). This may have led to differences in caregiver strategy use between the two studies. When comparing the rate per minute of strategy use with coaching, Dyads 1 and 2 used contingent modeling of targets and proximal targets at a similar rate per minute (4.2 and 4.1, respectively) as the two dyads in the N. S. Pak et al. (2024) study (5.4 and 4.1). Dyad 3 used contingent modeling at a lower rate (1.9 times per min). The rate of expansions was much lower in the current study (0.03–0.15 per min) compared to the previous study (0.34–0.52 per min). The rate of high-quality time delays was also lower in the current study (0.09–0.24 per min) compared to the previous study (0.26–0.37 per min).

When combined with fewer weekly visits, the shorter sessions likely resulted in insufficient coaching time for caregivers to master the strategies. A less drastic reduction in coaching time (e.g., 200–300 min per dyad) may have provided a better balance of feasibility and effective learning. Missed visits also sometimes resulted in multiple-week gaps between visits, especially for Dyads 3 and 4. Reasons for missed visits varied, but they included illness, unpredictable work schedules, and visits to family out of the country. This lack of consistency could have made it more difficult to recall strategies between sessions and integrate them into their habitual and daily interactions. Finally, this approach created more transitions within each session that could have affected child engagement. Overall, the reduced frequency and duration of coaching sessions necessitated procedural differences in the current study compared to previous studies, which may have contributed to the weaker effects.

Low rates of children’s spoken language and AAC use combined with short intervention sessions may help explain findings related to caregiver data, especially the lack of effects for Tier 2 strategies for Dyads 1 and 3. First, in some cases, caregiver rates of contingent modeling followed the same pattern as child rates of social communication (see Dyad 1 contingent modeling and child communication, Figure 3 and Figure 7). This makes sense, given that one aspect of contingent language modeling involves responding to what the child communicated. When the child communicates infrequently, caregivers have fewer natural opportunities to model language in response to the child.

Second, children’s low rates of language use may have led to insufficient opportunities for caregivers to practice and master expansions (Bailey et al., 2024). In the N. S. Pak et al. (2024) study, both children who completed the study had no spoken language at baseline; however, the children began producing speech output on AAC devices during the study. This provided caregivers with the opportunity to expand the children’s utterances. In the current study, only one caregiver used aided AAC modeling with her child, and the child did not begin independently using the device to communicate during the study. He did make progress in other ways that were observed informally and reported in mini-interviews, such as attending more frequently to the device. Recent meta-analytic findings indicate that child AAC use is related to the effectiveness of NDBIs in studies with autistic children (Pope et al., 2024). This finding reflects the responsive, contingent use of modeling and expansions as a core feature of NDBIs in general, and specifically as taught in the EMT en Español strategies. In the current study, the amount of aided AAC modeling and the specific communication apps varied, making it difficult to draw conclusions about effects of AAC on child communication. Future studies with this population need to place greater emphasis on aided AAC modeling and caregiver support with AAC to support children in learning to use this communication modality (Kent-Walsh et al., 2015). Latina mothers of children with developmental disabilities including autism have reported interest in more training using AAC (De Leon et al., 2023; McGuire et al., 2025). The range of potential perspectives among Latina mothers about AAC use should be further explored.

Other factors may also have affected caregiver opportunities to expand child utterances. Child 1 in the current study sometimes used many words during an observation, but they were often in English and did not provide optimal opportunities for the caregiver to use expansions in her native language. In earlier studies, a broad definition of expansions was adopted (responding to any child output by adding words to their utterance while attempting to maintain the child’s intent; N. S. Pak et al., 2024). In the current study, we adopted a stricter definition of expansions requiring caregivers to expand utterances to complete and grammatical sentences so that the strategy would not overlap with contingent target modeling. For example, if the child vocalized and pointed to a ball and the parent responded with “la pelota”, this was coded as contingent modeling and not as an expansion like in prior studies. The data indicate that expansions as defined in the current study may not be a useful or effective strategy to teach all families of autistic toddlers. Expansions may require a broader definition to accommodate the varying levels of child communication at baseline. They also may be more helpful to teach when families are more fluent in foundational strategies (e.g., contingent target language modeling) and when children have larger expressive vocabularies and higher rates of verbal communication (with or without AAC).

For Dyad 2, there was covariation between Tier 1 and Tier 2 data. We observed an increase in contingent proximal target-level modeling when target-level modeling was introduced. This indicates that the two strategies were not independent. These findings align with what was observed in the N. S. Pak et al. (2024) study with one of the dyads. In future studies, multiple baseline designs across behaviors may not be an appropriate design for testing these sets of intervention strategies with children who are not yet using symbolic language.

Child communication was our secondary dependent variable. In general, there were no clear patterns aligning with phase changes; Child 1, however, demonstrated an increase in gestures when his mother was taught time delays. It is possible that her greater focus on creating opportunities for the child to request during these observations resulted in more opportunities for the child to gesture. Overall, it is likely that the lower intensity of coaching or other procedural study features resulted in too few opportunities to see changes in child communication in relation to caregiver behavior.

### 4.1. Limitations and Future Directions

One limitation in the current study is that we did not meet our recruitment goal, and one family was unable to complete the study. Another limitation was that the strategies taught to the caregivers varied based on child communication skills. For example, the target behaviors for Dyad 2 in Tier 2 were contingent modeling of proximal targets, while they were expansions for Dyad 1. Although this complicates our interpretation of the graphs, we felt it was necessary for individualizing the intervention. Similar studies have also selected caregiver strategies based on participant characteristics (Lane et al., 2025).

A third limitation was that the intervention was delivered by the first author who is not Latina and a non-native Spanish-speaker. The strengths of the study are that the intervention was delivered in Spanish and the intervention itself has been culturally adapted. In practice, access to clinicians who share languages, cultures, and appropriate clinical training continues to be a workforce issue (American Speech-Language-Hearing Association, 2026). It is still possible that language or cultural barriers influenced intervention delivery. The inclusion of bilingual *and* bicultural clinicians has been reported by many families to be a major value in autism intervention studies (e.g., Pickard et al., 2024). Train the trainer studies involve service provision by *promotoras* (Magaña et al., 2017) or bilingual graduate student clinicians (McGuire et al., 2025) who share a linguistic and cultural background with the families and are given training in the clinical components of language intervention and coaching. This could be an alternative model for more thoroughly addressing the *persons* dimension of cultural adaptation as well as addressing needs in the workforce (Bernal et al., 1995; Lee et al., 2024). Moreover, intervention delivery for all dyads by a single therapist limits our ability to infer the feasibility of this intervention for other therapists, such as bilingual graduate students.

A fourth limitation is that modeling and coaching was only conducted in the context of play, due to the brief duration of sessions. Latino parents have reported finding it more acceptable to teach across a variety of activities, rather than just play (L. M. Cycyk & Huerta, 2020). Furthermore, in the current study, caregiver strategy use in generalized contexts (i.e., without coaching in different activities) was generally less frequent than strategy use in intervention contexts (i.e., with coaching during play). Although ways to use the strategies throughout the day between visits were consistently discussed with families in the review portion of the visits, TMCR instruction across activities may be necessary for many families to facilitate generalization of learned skills.

### 4.2. Implications and Conclusions

This research leads to several implications. First, reduced visit frequency and multiple TMCR cycles per visit may not be the ideal intensity or format to support learning of EMT en Español Para Autismo strategies. In our attempt to reduce burden on families, we restricted dosage to an extent that likely weakened the potential for positive effects, especially for children with low rates of communication (Dyads 2 and 4). We also created visit structures with multiple transitions which tend to be difficult for autistic children (American Psychiatric Association, 2022). While taking breaks according to the child’s engagement state is a useful strategy for some children, in the current study, these adult-led transitions were disruptive and resulted in the need to re-engage the child in play. Future studies should continue to explore innovative and adaptive approaches to increasing access to culturally adapted NDBIs for a wide range of Latino Spanish-speaking families with autistic children, including those who may have limited time and resources for participating in intensive weekly therapy. There are also Latino families who want or require more therapy time (DuBay, 2022; N. S. Pak et al., 2024). Delivering most or all visits via telehealth to allow for more flexible scheduling may be another option (e.g., Bailey et al., 2024). More research is needed to identify the appropriate balance between caregiver preference, feasibility, and intervention dosage of EMT en Español Para Autismo.

Relatedly, one visit per week with 10–20 min of direct therapy (modeling and coaching) with the child did not lead to observable changes in child communication skills. Given that caregivers in the current study valued modeling the most of the TMCR components, and one caregiver expressed the need for longer direct therapy time, future models with continued direct therapist–child intervention sessions may be most appropriate. This would ensure a higher dosage of active ingredients of the intervention for the child without necessarily increasing caregiver burden.

Third, in the current study, coaching was limited to play interactions, and generalization effects were limited. In research and practice, coaching across multiple contexts will be important to ensure access to communication gains across multiple meaningful family activities (Peredo, 2016). In a randomized trial (*n* = 77) testing the effectiveness of a therapist + caregiver-implemented model of EMT en Español with Spanish-speaking Latino children with developmental language disorder, there were moderate to strong, significant effects of coaching on parent use of contingent modeling and on children’s language outcomes (Peredo et al., 2026). In this study, caregivers received 1 weekly TMCR coaching session and their child additionally received a weekly 35 min therapist + child session. The intervention similarly lasted 5 months but also included coaching in routines (Peredo et al., 2026). Thus, compared to the current study, it reduced the burden on caregivers while providing more direct intervention to the child. This intervention model may be more effective for children with autism and acceptable to families with competing demands.

Fourth, given consistent feedback from Latina caregivers that reducing instructions and questions is not culturally congruent, future EMT en Español Para Autismo iterations should support caregivers to use multiple types of utterances (including instructions and questions) in ways that optimally support child communication and learning.

## Figures and Tables

**Table 1:** Key differences in study features.

Study Feature	N. S. Pak et al. (2024)	Current Study
Number of teach–model–coach–review cycles per visit	1	1–2
Duration of coaching per visit	15 min (10 min coded)	5–10 min
Frequency of 1 h visits	Up to 3 per week	1 per week
Overall duration of coaching for each dyad ^a^	405–495 min	95–125 min
Contexts for coaching	Play, book-reading, routines	Play
Generalization observation context	Uncoached interactions in intervention contexts (play, book-reading, routines)	Uncoached interactions in non-intervention contexts (book-reading, routines)

^a^ Actual coaching time for participants that completed the study.

**Table 2:** Participant characteristics.

	Dyad 1	Dyad 2	Dyad 3	Dyad 4
Completed the study?	Yes	Yes	Yes	No
**Child**				
Autism diagnosis	Yes	Yes	Yes	No
Age at baseline (months)	41	47	33	35
Gender	Boy	Boy	Girl	Boy
Number of different words ^a^	Spanish: 5	Spanish: 0	Spanish: 17	Spanish: 1
	English: 25	English: 0	English: 1	English: 0
	Conceptual: 27	Conceptual: 0	Conceptual: 18	Conceptual: 1
PLS-5 Spanish EC standard score	61	50	63	50
Language Exposure—weekday	57% Spanish 43% bilingual (Spanish and English)	85% Spanish 15% English	100% Spanish	57% Spanish 43% English
Language Exposure—weekend	100% Spanish	100% Spanish	100% Spanish	100% Spanish
Services	Early intervention until age 3 (Sp); began ABA during the study (Sp)	Early intervention (Sp/En); speech therapy (En)	Early intervention (Sp); Preschool (NA)	Early intervention (Sp/En); began preschool during the study (En)
**Caregiver**				
Relationship to child	Mother	Mother	Mother	Mother
Age category (years)	40–49	30–39	20–29	30–39
Education level	Bachelor’s degree	Some college	High school	Bachelor’s degree
Household Income-to-Needs Ratio ^b^	2.25	1.13	1.24	0.75
**Planning**				
Individualization based on FVAI and Child Observation Protocol ^c^	Use aided modeling; incorporate animals and art in play activities; use small chair and limit external sounds to help focus	Use aided modeling; incorporate fine motor activities (e.g., blocks) and other preferred toys (e.g., cookies); monitor sensory needs (e.g., dim lights)	Spoken modeling only; use highchair for some activities to support focus; incorporate sensory play activities (e.g., slime) and a variety of play activities	Spoken modeling only; incorporate activities involving water; frequent breaks because engagement with toys was typically brief

Note. Sp = Spanish. En = English. EC = Expressive Communication. NA = not available. FVAI = Family Values and Activities Interview (Peredo, 2016). PLS-5 Spanish = Preschool Language Scales, 5th edition Spanish (Zimmerman et al., 2012). ^a^ Across two 20 min semi-structured language samples. Dyad 4 only participated in one language sample. ^b^ Annual household income divided by the 100% threshold for the number of people in the family according to the 2025 poverty guidelines (U.S. Department of Health and Human Services, Office of the Assistant Secretary for Planning and Evaluation, 2025). ^c^ During the therapist–child intervention sessions.

**Table 3:** Effect size estimates.

Dyad	Tier 1	Tier 2	Tier 3
1	Contingent modeling	Expansions	Time delays
	Tau-U_BC_ = 1.00	Tau-U_BC_ = −0.18	Tau-U_BC_ = 0.50
	SE = 0.01	SE = 0.18	SE = 0.30
	CI: [1.00, 1.00]	CI: [−0.57, 0.29]	CI: [−0.12, 0.83]
2	Contingent target-level modeling	Contingent proximal target modeling	Time delays
	Tau-U_BC_ = 1.00	Tau-U_BC_ = −0.40	Tau-U_BC_ = 0.44
	SE = 0.01	SE = 0.21	SE = 0.19
	CI: [1.00, 1.00]	CI: [−0.70, 0.06]	CI: [−0.03, 0.74]
3	Contingent modeling	Expansions	Time delays
	Tau-U_BC_ = 1.00	Tau-U_BC_ = 0.21	Tau-U_BC_ = 0.60
	SE = 0.01	SE = 0.15	SE = 0.25
	CI: [1.00, 1.00]	CI: [−0.19, 0.55]	CI: [0.04, 0.86]
4	Contingent target-level modeling	-	-
	Tau-U_BC_ = 1.00		
	SE = 0.05		
	CI: [1.00, 1.00]		

Note. Tau-U_BC_ = baseline corrected Tau-U. Tau-U of 0.20 = small, 0.20 to 0.60 = moderate, 0.60 to 0.80 = large, and above 0.80 = large to very large effect size, depending on context (Vannest & Ninci, 2015).

## Data Availability

The data presented in this study are available on request from the corresponding author. Some data are not available to protect participant identities.

## Supplementary Materials

The following supporting information can be downloaded at https://www.mdpi.com/article/10.3390/bs16081457/s1, Supplementary File S1: Child Observation Protocol. Supplementary File S2: Cultural Adaptation Examples Using Lee et al.’s (2024) Checklist. Supplementary File S3: Child Average Word Use Across Tiers.
