# Supplementary material for: Caregiver-Implemented Lower-Intensity EMT en Español Para Autismo: A Single Case Design Study
Source: Behav Sci (Basel). 2026 Aug 21;16(8):1457. doi: 10.3390/bs16081457 (PMC13509933; doi:10.3390/bs16081457)
Supplement: Supplementary file 1 [file behavsci-16-01457-s001.zip › Supplementary File S1.pdf]

Supplementary File S1
Child Observation Protocol

Complete this form while observing the interventionist use EMT en Español Para Autismo strategies directly with the child. The observation can be in person or from video.

Study ID: __________________

Date of session: __________________

Date of observation: __________________

Duration of observation (min): __________________

Observer name: __________________

Mode of observation (circle one): in person / video

Behavior Support

If the therapist used any of the following strategies, check the box and record observations:

**Strategy**	**Describe use of strategy and child response (include time stamp)**
☐ Timer	
☐ Visual schedule	
☐ Taking breaks	
☐ Other strategy: __________________	
☐ Other strategy: __________________	

☐ Other strategy: ____________________	

Response to Intervention

Describe the child's response to use of the following strategies:

Strategy	Describe use of strategy and child response (include time stamp when appropriate)
Environmental Arrangement (e.g., limiting distractions, limiting space to wander)	
Modeling Target and Proximal Target Language	
Matched Turns	
Imitating Child Actions	
Recasts/Expansions	
Time Delays	
Milieu Prompts	

Child Engagement and Preferences

List activities the interventionist did with the child and the child's engagement in each:

Activity	Did the child seem to enjoy this activity?
	☐ Yes<br>How long? __ min<br>How could you tell?<br><br>☐ No<br>How could you tell?
	☐ Yes<br>How long? __ min<br>How could you tell?<br><br>☐ No<br>How could you tell?
	☐ Yes<br>How long? __ min<br>How could you tell?<br><br>☐ No<br>How could you tell?
	☐ Yes<br>How long? __ min<br>How could you tell?<br><br>☐ No<br>How could you tell?
	☐ Yes<br>How long? __ min<br>How could you tell?<br><br>☐ No<br>How could you tell?
	☐ Yes<br>How long? __ min<br>How could you tell?<br><br>☐ No<br>How could you tell?

Any other comments about the session you observed:
