# Supplementary material for: Caregiver-Implemented Lower-Intensity EMT en Español Para Autismo: A Single Case Design Study
Source: Behav Sci (Basel). 2026 Aug 21;16(8):1457. doi: 10.3390/bs16081457 (PMC13509933; doi:10.3390/bs16081457)
Supplement: Supplementary file 1 [file behavsci-16-01457-s001.zip › Supplementary File S2.pdf]

Supplementary File S2

Cultural Adaptation Examples Using Lee et al.'s (2024) Checklist

**Cultural Adaptation Checklist Dimension**	**Examples in the Current *EMT en Español Para Autismo* Study**
Language	All written materials are provided in Spanish.<br><br>Study related written materials (consent forms, recruitment materials) were backward and forward translated by bilingual staff to ensure accuracy.<br><br>The therapist checks with the family every session for dialect-specific and family-specific vocabulary to be used.<br><br>Comprehension checks are conducted regularly throughout the session and especially during the teach and review portions.
Persons	The adaptation process involved consultation with Latina Spanish-speaking caregivers of autistic children (Pak et al., 2024).<br><br>Therapists are bilingual.<br><br>Caregivers provide feedback on the intervention during and after implementation via mini-interviews and the exit interview.
Content	Child language targets align with typical Spanish language developmental sequences.<br><br>The adaptation process involved consultation with Latina Spanish-speaking caregivers of autistic children (Pak et al., 2024).<br><br>Strategies were culturally adapted to align with common Latino values. For example, caregivers were taught to select activities and then follow children's interests within each activity, rather than simply following the child's lead (Peredo et al., 2022).

Goals	Target level language is based on typical child Spanish language development and the child's baseline language skills.<br><br>The FVAI addresses the caregiver's goals for the child's participation and communication.<br><br>Social validity is assessed via interviews.<br><br>Communication measures allow for children to demonstrate language skills bilingually.
Methods	Sessions have cyclical Teach-Model-Coach-Review structure to reduce weekly visits.<br><br>The therapist communicates with the families in their preferred language(s).
Contexts	All assessments and intervention activities occur in the families' homes or other preferred community locations.<br><br>The therapist was as flexible as possible with scheduling sessions to support the family's ability to continue to participate in the study.<br><br>The FVAI addresses contextual factors (time constraints, etc.) relevant to access to services and participation in intervention.
Process	The cyclical and reduced intensity iteration of the EMT en Español Para Autismo intervention was implemented in response to retention issues in the previous iteration (Pak et al., 2024).
