# Supplementary material for: Caregiver-Implemented Lower-Intensity EMT en Español Para Autismo: A Single Case Design Study
Source: Behav Sci (Basel). 2026 Aug 21;16(8):1457. doi: 10.3390/bs16081457 (PMC13509933; doi:10.3390/bs16081457)
Supplement: Supplementary file 1 [file behavsci-16-01457-s001.zip › Supplementary File S3.pdf]

Supplementary File S3

Child Average Word Use Across Tiers

Child	Measure	Baseline	Tier 1	Tier 2	Tier 3
1	Total Words	4.7 (0–14)	7.8 (1–37)	2.8 (1–4)	4.0 (1–6)
	Different Words	3.2 (0–8)	4.0 (1–14)	2.3 (1–4)	3.8 (1–6)
2	Total Words	1.7 (0–5)	0.9 (0–3)	0.8 (0–3)	0
	Different Words	1.5 (0–4)	0.9 (0–3)	0.8 (0–3)	0
3	Total Words	12.0 (1–43)	10.9 (0–32)	8.6 (0–32)	13.2 (3–29)
	Different Words	8.8 (1–33)	7.3 (0–15)	5.3 (0–17)	8.4 (1–28)
4	Total Words	0.2 (0–1)	0.5 (0–2)	–	–
	Different Words	0.2 (0–1)	0.5 (0–2)	–	–

Note. Values represent the mean number per 5 min coached session with ranges in parentheses.
